# X-ray photoelectron and infrared spectroscopies of quartz samples of contrasting toxicity

**DOI:** 10.1186/1476-069X-8-S1-S4

**Published:** 2009-12-21

**Authors:** Stephen M Francis, W Edryd Stephens, Neville V Richardson

**Affiliations:** 1 School of Chemistry, University of St Andrews, Purdie Building, North Haugh, St Andrews, Fife KY16 9ST, UK; 2 School of Geography & Geosciences, University of St Andrews, Irvine Building, North Street, St Andrews, Fife KY16 9AL, UK

## Abstract

An exploratory XPS and FTIR investigation of the surfaces of bulk quartz powders widely used in toxicological studies (DQ12 and Min-U-Sil 5) was carried with the aim of correlating surface features with toxicity as reflected by indicators of biological response. Some patches of amorphous silica were identified as well as varying amounts of calcium but none of these features correlated with biological response. No evidence of widely-quoted surface silanol (SiOH) structures was found in this investigation and the possibility that FTIR artefacts have been previously misidentified as silanol structures is discussed.

## Background

Quartz dust of respirable size is associated with silicosis and other lung diseases [1] following inflammatory response to the inhaled particles [2]. The mineral surface is implicated as occlusion of the surface of quartz dusts largely eliminates the response [3] and variations in biological response are frequently attributed to variations in surface reactivity of quartz [4]. The mechanism of the effect is poorly understood and various aspects of the mineral surface have been implicated including surface free radicals, surface impurities such as Fe, and surface silanol groups [5-7]. This study examines surface chemical characteristics of bulk quartz powders using Fourier transform infrared spectroscopy (FTIR) and X-ray photoelectron spectroscopy (XPS) with the aim of identifying structures potentially relevant to the toxic effect. Two quartz samples associated with different biological response were investigated. We searched for impurities and surface silanol structures with the aim of identifying any relationship to differences in known biological response for the same powders.

## Methods

Samples chosen for study were Min-U-Sil 5 and DQ12, both already well characterised for particle size, surface area and biological response [8-10]. No pre-treatment or particle size separation was performed on these samples therefore all results are indicative of the as-received samples. DQ12 is regarded as particularly toxic [8-10] and the spectra for this sample and the apparently less toxic Min-U-Sil 5 are presented below. X-Ray photoelectron spectroscopy (XPS) data were collected on a VG ESCALAB II instrument with un-monochromated Al Kα radiation. The samples were prepared in pure form by adhesion to double sided, electrically conducting tape. Pass energies, scan sizes and data collection times were adjusted to give the required signal to noise ratio. Analysis of the collected data was performed using the CasaXPS software. Infrared spectra were obtained using a Nicolet FTIR instrument in transmission mode on samples prepared as either a dilute solution in KBr and pressed into 10 mm diameter pellets, (a pure KBr disc was used as a background reference) or spread on top of a 5 bounce ZnSe ATR crystal. This ATR method is favoured as it operates on the pure samples and avoids any moisture content within the KBr which can cause mis-cancellation problems. In all experiments a DGTS detector was employed which is essential when performing experiments involving water adsorption (see Discussion).

## Results

**Figure 1:**
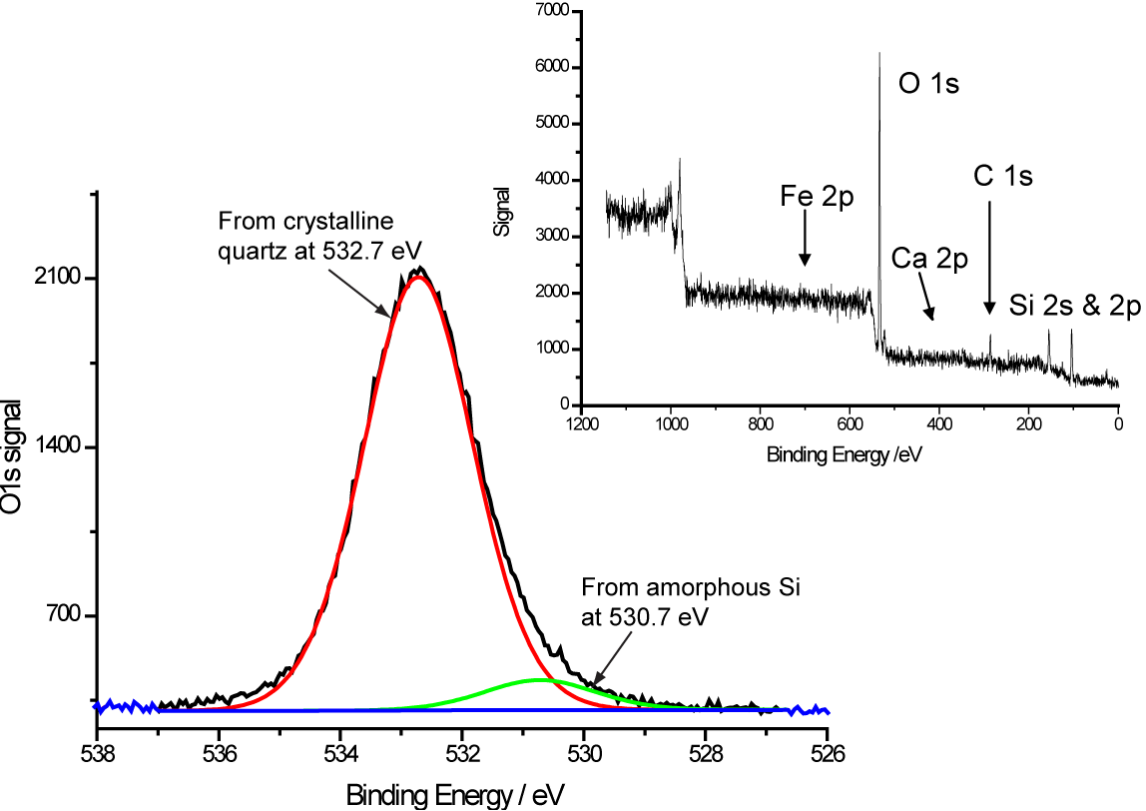
**XPS spectra of DQ12 crystalline quartz**. Oxygen 1s XPS peak from DQ12 crystalline quartz showing no evidence of a Si-OH peak at 531.9eV. The insert is the wide scan of the same sample indicating very little surface contamination.

The XPS spectra indicated the presence of very little contamination in any sample (Figure 1). There was no evidence of any Fe-bearing species. Carbon, ubiquitous on practically all materials exposed to ambient conditions, was present on all samples analysed but in smaller quantities than might be expected given the natural origin of the samples. Detailed analysis of the O1s emission peak showed no evidence of silanol species (Si-OH) on the surface but does indicate the presence of a small amount of non-crystalline, amorphous SiO_2_. It was not possible to identify whether these were discrete amorphous particles or amorphous patches on crystalline particles. None of the observed surface contaminants or structures correlate with known differences in biological response.

**Figure 2:**
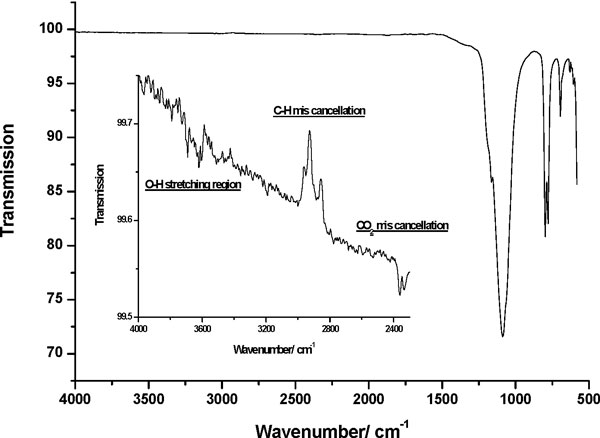
**FTIR spectrum of Min-U-Sil 5 crystalline quartz**. The IR spectrum of Min-U-Sil 5 is shown, collected using an ATR attachment combined with a DTGS detector. The O-H stretching region centred around 3500 cm^-1 ^is shown on an appropriate scale. No evidence of a significant absorption band can be seen in this region.

**Figure 3:**
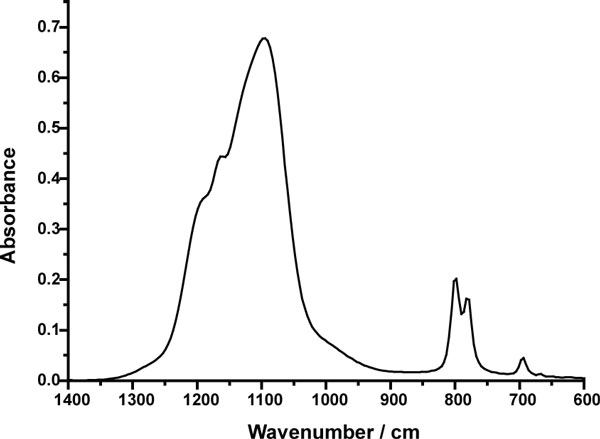
**FTIR spectrum of DQ12 crystalline quartz**. A small region of the FTIR spectrum of the DQ12 quartz sample is shown. The expected silanol (hydrogen bonded Si-O-H) band at ~900 cm^-1 ^is absent.

The FTIR spectra of Min-U-Sil 5 obtained using the ATR method is shown in Figure 2. The insert shows that even when the O-H stretching region is displayed on an appropriate scale, no significant absorption is observed. Figure 3 shows the IR absorbance spectrum of DQ12 in the region of the bulk vibrations. This spectrum is representative of many silica samples investigated and is devoid of bands around 900 cm^-1 ^that can be associated with hydrogen bonded Si-O-H (silanol) species.

## Discussion

The work of Fubini et al [5] explicitly specifies the use of a liquid nitrogen cooled MCT detector, the same type of detector is assumed in the work of Pandurangi et al. although this is not specified [10]. An inherent problem with this type of detector is the condensation of water vapour and subsequent water ice formation on the cold detector window as a function of time. Indeed [10] shows spectra for two types of quartz samples where the O-H stretching band at ~3400 cm^-1 ^is comparable in magnitude to that of the bulk Si-O stretching bands at ~1100 cm^-1^. This considerably exceeds what might be expected for surface H_2_O or OH species. No spectra showing the bulk bands are given in Fubini et al [5] and therefore direct comparison is not possible. Detectors of this type should be avoided when studying water/solid systems as this inherent experimental artefact will always obscure any information pertaining to the adsorption of water on the sample surface. Any data relating to this band should always be interpreted cautiously when using an MCT detector. In all studies presented here a DTGS detector was used, although this type of detector is not as sensitive as an MCT detector it does not suffer from this experimental disadvantage. In view of this experimental obstacle the silanol band at ~900 cm^-1 ^is taken to be a better measure of surface hydroxyl concentration.

FTIR spectra suggest that silanols are not present to any great degree on the surfaces these quartz powders, and this is in agreement with the findings from XPS. Indeed, a sample of Min-U-Sil 5 was made into a thick paste with deionised water and dried under mild conditions (24 hours at 120°C) and showed no discernable difference from the untreated sample. Silanols are predicted to be widespread on hydrolysed surfaces of quartz [11] and their apparent absence is difficult to explain. Thus it was not possible to correlate silanol density with the contrasting biological responses of these two powders. Due to the nature of the toxicity tests in aqueous solution, the presence of surface silanols during the toxicity tests certainly cannot be ruled out and indeed would be expected from theoretical considerations. Pandurangi et al. [12] identified O-H bands in FTIR spectra around 3750 cm^-1 ^and related these to their haemolysis results suggesting that the correlation was relevant to the cytotoxicity of respirable silica particles. Our work does not support this finding given the absence in our experiments of the corresponding O-H bands at ~900 cm^-1 ^and we suggest that this relationship requires further investigation. FTIR investigations searching for surface hydroxyl species or physisorbed water is unfeasible in aqueous solution as phase modulation techniques are inappropriate for this type of system.

The XPS data does however indicate the presence of amorphous silica within the samples, this may be a fertile area for further investigation as the nature of this amorphous silica is unknown at this time. It may be distinct from or present as an integral or surface component of the crystalline quartz.

## Conclusion

XPS and FTIR examination of quartz samples with varying biological response did not reveal any features of the particle surface chemistry that correlates with haemolysis or other indices of biological response. There is some evidence of amorphous silica and further investigation of this feature is necessary.

## Note

The peer review of this article can be found in Additional file 1.

## Competing interests

The authors declare that they have no competing interests.

## Authors' contributions

SMF carried out the FTIR and XPS studies. WES, SMF and NVR conceived of the study, participated in its design and coordination, and helped to draft the manuscript. All authors read and approved the final manuscript.  

## Supplementary Material

Additional file 1 Peer review. Click here for file
