# Supplementary material for: X-ray photoelectron and infrared spectroscopies of quartz samples of contrasting toxicity
Source: Environ Health. 2009 Dec 21;8(Suppl 1):S4. doi: 10.1186/1476-069X-8-S1-S4 (PMC2796500; doi:10.1186/1476-069X-8-S1-S4)
Supplement: Additional file 1 — Peer review. [file 1476-069X-8-S1-S4-S1.doc]

| Article ref no. | A1471-2105X SZ S4 |
| --- | --- |
| Title | **X-ray photoelectron and infrared spectroscopies of quartz samples of varying toxicity.** |
| Author(s) | **Francis et al.** |

Title now changed to **X-ray photoelectron and infrared spectroscopies of quartz samples of contrasting toxicity to better reflect the content.**

# Ref#1

**General comments:**

This brief article addresses an important topic of concern relating to health implications associated with quartz particles.  I believe, however, that to warrant publication further discussion is required to render it a stand-alone article.

**Major compulsory revision:**

1. The paper relates to bulk materials.  These are likely to include a broad range of sizes and shapes which are likely to be significant to the toxicology.  Could the toxicology simply relate to a specific sub-population of particles?

Quite probably. This is a very short paper in which we chose to focus on the surfaces of bulk powder but we do indeed suspect surface area to be a key feature in the toxicity of quartz, and the sub micron subset is likely to be the biggest contributor. New data generated since the first submission and to be published elsewhere will support this. Of course all subsets are represented in the bulk powder and, unless they are very small in mass terms, they should provide signals of the presence of implicated structures such as silanols.

2. Further to the above comment, no information on variability in analyses is provided.  From the above, I would estimate variability to be high.  Semi-quantitative assessments of the various materials would have been preferable.

We hope this point is accommodated in part by providing more graphs showing in detail the variability in the important FTIR signal.

3. In biological environments, ionic, free radical h.  Semi-quantitative assessments of the various materials would have been pre and enzymatic interactions are likely to influence reactivity/toxicity.  Also, how might pulmonary surfactants alter particulate behaviour?  These important factors are salient and warrant discussion.

We have now made it clear that we are dealing with anhydrous experiments and our toxicology results are no longer discussed, just those in the literature. That said, this issue is important and will be discussed at length in a later paper.

**Ref#2**

**General comments:** This is a short paper describing XPS and IR data for a set of quartz samples of varying toxicities. This is a well-researched area, and the authors quote some of the key previous publications in the manuscript. I have major concerns regarding the content of this manuscript, which may be due to lack of sufficient information rather than the soundness of the data; however I feel resubmission is necessary.

**Major compulsory revisions:**

1) A central conclusion of the paper is lack of evidence for silanol structures. However, key information about the way data collection was carried out is missing, so I cannot assess whether this conclusion is valid. Specifically it is essential to describe the powders studied (e.g. particle size and size distribution), what pretreatment, if any, was carried out prior to FTIR and XPS data collection and what were the exact conditions of analysis. The authors state “… the presence of surface silanols during the toxicity tests cannot be ruled out and indeed would be expected from theoretical considerations”. This is absolutely correct, so there should be at least some attempt to justify why silanol groups are not seen in the present study. Specifically for FTIR data, the authors claim absence of silanol on the basis of the absence of a peak at ~900cm^-1^; however, according to Pandurangi et al (full reference quoted in the manuscript) this is only a “weak kink”. The most characteristic feature is a sharp band at 3745cm^-1^; have the authors looked for this feature? I believe this region may be affected by scattering of the IR radiation above certain particle size, so they may have had problems acquiring the data, but this needs to be declared.

Furthermore, if the lack of detectable silanols is due to the samples being studies in air, rather than water, and if the presence of silanols in aqueous toxicity tests is what is believed to correlate with toxicity then what is the value of the present study? Perhaps aqueous methods should have been used instead (e.g. ATR-FTIR).

This set of issues has become the main focus of the redrafted manuscript and we believe that every point raised here has now been addressed and the paper significantly improved as a result.

2) The presence “patches of amorphous silica”, the only other major conclusion of this study, should be explained and justified further. The authors could be helped a lot by introducing a better description of their samples, as mentioned earlier. For example, are these patches structural surface features (as the authors appear to imply) or could they be loose amorphous silica nanoparticles mixed in their powders?

We have elaborated a little on this but it does require further experimentation that we have not yet conducted.

3) The study is about “a range of bulk quartz powders”, only two of which are mentioned by name, and only one presented in graphs. It is essential to include a table showing all the powders tested, and to include key information about each one (name, source, crystal structure, particle size and size range).

We have now reduced the paper to a consideration of the two named samples and quoted literature studies where all the information requested is presented.

4) The presence of carbon needs to be discussed further, i.e. what is the source? Is it just surface contamination or is it structural? It is not clear to me why “it is expected given the nature of the samples.”

We have no way of knowing the source of carbon. The comment merely reflects the experience of many years XPS analysis of synthetic and natural samples. Natural samples often contain higher levels of carbon than usually found in synthetic samples, presumably reflecting atmospheric and possibly other environmental contamination sources.

5) p.4, 1^st^ sentence: “hydrolysed” I believe you mean “hydrated”.

Hydrolysed is actually correct in this context

6) p.4, 14^th^ line: define “Fenton-type reactions”.

Discussion of Fenton-type reactions has been removed in the revised version.
